# Causal effect of thyroid cancer on secondary primary malignancies: findings from the UK Biobank and FinnGen cohorts

**DOI:** 10.3389/fimmu.2024.1434737

**Published:** 2024-09-26

**Authors:** Zhengshi Wang, Youlutuziayi Rixiati, Chengyou Jia, Yong Xu, Zhiqiang Yin, Junwen Huang, Jiaqi Dai, Yun Zhang

**Affiliations:** ^1^ Department of Breast and Thyroid Surgery, Shanghai Tenth People's Hospital, School of Medicine, Tongji University, Shanghai, China; ^2^ Shanghai Center of Thyroid Diseases, Shanghai Tenth People's Hospital, School of Medicine, Tongji University, Shanghai, China; ^3^ Department of Pathology, Fudan University Huashan Hospital, Shanghai, China; ^4^ Department of Nuclear Medicine, Shanghai Tenth People’s Hospital, Tongji University, Shanghai, China; ^5^ Department of Laboratory, Yueyang Hospital, Hunan Normal University, Yueyang, China

**Keywords:** thyroid cancer, GWAS, Mendelian randomization, bladder cancer, SNP

## Abstract

**Background:**

Existing epidemiological data indicated a correlation between thyroid cancer (THCA) and the risk of secondary primary malignancies (SPMs). However, the correlation does not always imply causality.

**Methods:**

The Mendelian randomization (MR) analyses were performed to investigate the causal relationships between THCA and SPMs based on international multicenter data. Odds ratios (ORs) with 95% confidence intervals (95% CIs) were calculated. The Cancer Genome Atlas (TCGA) was used to explore potential mechanisms shared by THCA and bladder cancer (BLCA).

**Results:**

Summary datasets of genome-wide association studies (GWAS) on 30 types of cancers were obtained from the United Kingdom Biobank (UKB) and FinnGen database. Meta-analysis of the UKB and FinnGen results revealed that THCA was significantly positively correlated with BLCA (OR = 1.140; 95% CI, 1.072-1.212; P < 0.001). Four genes, including WNT3, FAM171A2, MLLT11, and ULBP1, were identified as key genes shared by both TCHA and BLCA. Correlation analysis indicated that THCA may increase the risk of secondary BLCA through augmentation of N2 neutrophil infiltration.

**Conclusions:**

This study showed that THCA was causally related to BLCA. It is recommended to conduct more rigorous screenings for BLCA during the follow-up of THCA patients.

## Introduction

Thyroid cancer (THCA) is the most common malignancy in the endocrine system (1). Surgery is the primary treatment approach for THCA, usually accompanied by postoperative radioactive iodine (RAI) and endocrine therapy (2, 3). There are two major pathological types: papillary, follicular, medullary and anaplastic THCA (4). Papillary and follicular TCHA collectively refer to as differentiated THCA, which has a favorable prognosis with 10-year survival rate > 90% (5). The prolonged survival period in THCA patients may lead to the emergence of secondary primary malignancies (SPMs). Epidemiological findings revealed that previous diagnosis of THCA increased the risk of SPMs (6–9). However, there were some inconsistencies among epidemiological conclusions (10). On the other hand, epidemiological correlation doesn’t necessarily indicate causality. Therefore, it is necessary to investigate the true causality between THCA and SPMs.

Mendelian randomization (MR) analysis is a statistical method that uses genetic variants as instrumental variables to assess causal relationships. In comparison to observational or epidemiological studies, MR demonstrates excellent performance in inferring causality (11, 12). When the sample size is small, epidemiological approaches might not yield statistically significant outcomes. However, MR can assess the impact of genotypes randomly allocated in nature on phenotypes, allowing for the accurate determination of causal relationships between exposure and outcome (not mere correlation). Currently, more and more studies have been focusing on the causality between various biological factors and diseases using the MR method (13).

In the present study, we used the genome-wide association study (GWAS) summary datasets to investigate the causal relationships between THCA and common malignancies systematically. This would provide the basis for a more profound understanding of potential connections between diseases and better management of THCA patients.

## Methods

### Study design

**Figure 1:**
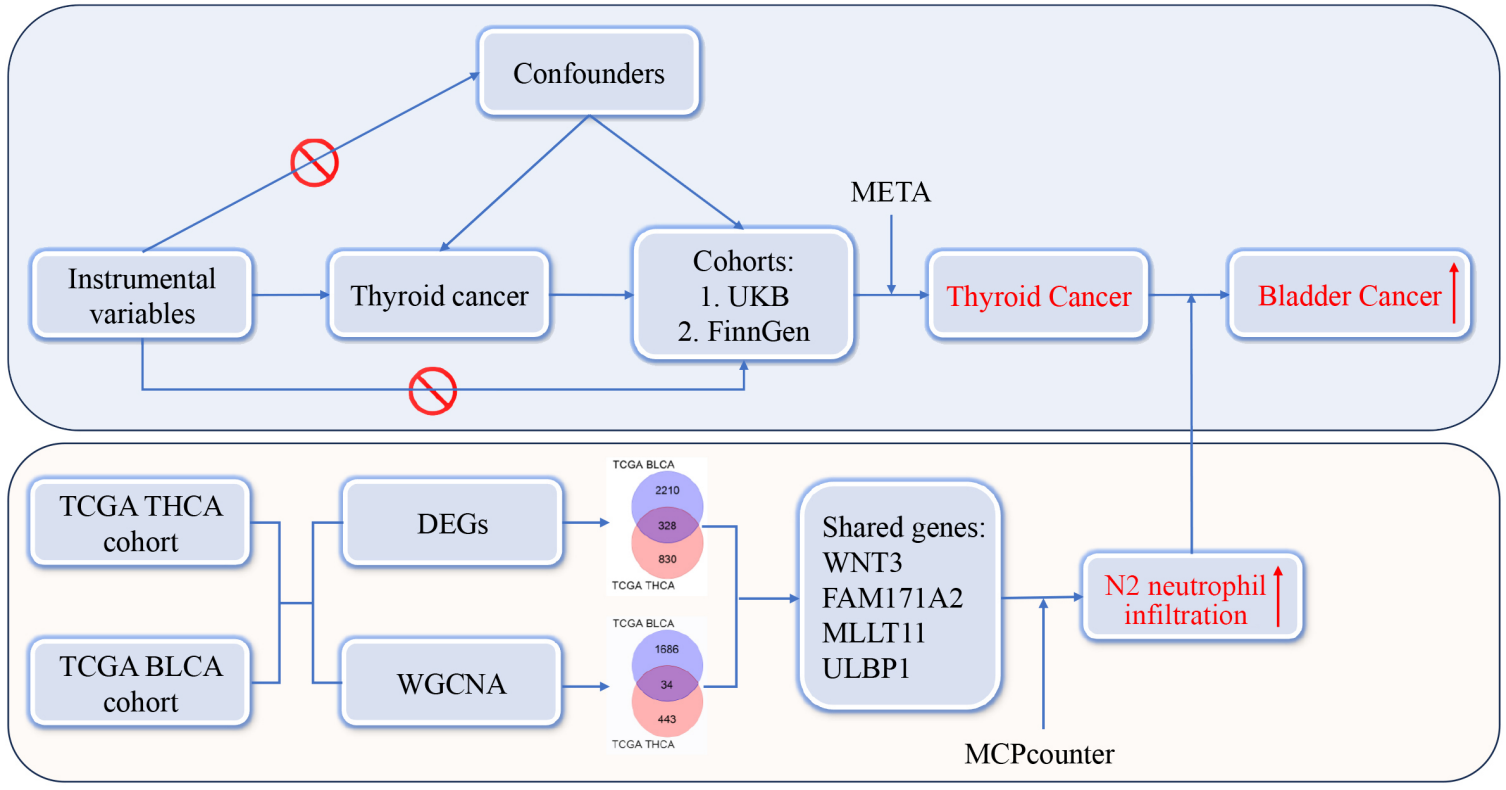
Design and analysis workflow.

The design and analysis workflow were shown in **Figure 1**. There were two sections in the present study. The first section was to investigate the relationships between THCA and SPMs. Instrumental variables employed in the study were single nucleotide polymorphisms (SNPs), which refer to the DNA sequence variations caused by changes in a single nucleotide at the genomic level. The exposure factor was THCA and the outcomes were common malignancies from the United Kingdom Biobank (UKB) (http://www.ukbiobank.ac.uk) and FinnGen database (https://www.finngen.fi/en). MR analyses were performed, and a final conclusion was reached by conducting a meta-analysis on the MR results from the UKB and FinnGen databases. Based on the comprehensive analysis in the first section, we found that THCA was significantly positively correlated with bladder cancer (BLCA). The second section was to search for possible mechanisms between THCA and BLCA. The Cancer Genome Atlas (TCGA) database (http://tcga-data.nci.nih.gov/tcga/) was used to obtain the bulk RNA-seq data of THCA and BLCA. Two different statistical approaches, including differentially expressed gene (DEG) and weighted gene co-expression network analysis (WGCNA), were used to identify shared genes between THCA and BLCA. Immunoinfiltration analysis was performed to identify possible mechanisms. The study was approved by the Institutional Review Board of Shanghai Tenth People’s Hospital. Data acquisition was completely dependent on public databases and informed consent was not required. The present study followed STROBE criteria.

### Exposure and instrumental variables

Initially, the GWAS summary datasets of THCA were obtained from the GWAS Catalog (https://www.ebi.ac.uk/gwas/downloads/summary-statistics). GWAS summary datasets refer to a comprehensive collection of big data generated from GWAS studies, typically encompassing statistical outcomes for millions of SNPs. However, it was difficult to identify stable and reliable instrumental variables due to the small sample sizes of THCA datasets. Thus, we used the THCA dataset from The Polygenic Score (PGS) Catalog (https://www.pgscatalog.org/, PGS Publication No.: PGP000262). There were 6,699 THCA cases and 1,613,655 controls, which were sufficient to identify effective and stable instrumental variables. The screening criteria of instrumental variables were as follows: (1) The genome-wide significance was set at a P-value of 5 × 10^–8^. (2) To ensure independence between instrumental variables, the clustering distance threshold was set at 10,000 kb and linkage disequilibrium correlation coefficient r^2^ was set to 0.001. (3) SNPs associated with confounding factors were removed. Common confounding factors include diabetic condition, hypertension, alcohol consumption, tobacco use, and body mass index (14–16). The exclusion of confounding factors improved the objectivity and accuracy of causal relationships. (4) SNPs with F-statistic greater than 10 were considered as strong instrumental variables. The F-statistic was estimated using the formula: F = (Beta/SE)^2^. Beta represents the estimated effect size of the relationship between the SNP and exposure, and SE denotes the standard error of the Beta value. (5) There was no correlation between instrumental variables and outcome. SNPs directly associated with cancer were eliminated and detailed list was presented in **Supplementary Table 1**.

### Pan-cancer GWAS data

GWAS summary datasets of common malignant neoplasms of eight human organ systems were obtained from the UKB and FinnGen database, including integumentary system, genital system, urinary system, nervous system, digestive system, respiratory system, circulatory system, and motor system. There was a total of 30 and 26 cancer types available in the UKB and FinnGen database, respectively. Detailed data of each cancer can be found in **Supplementary Tables 2**, **3**.

### MR analysis and meta-analysis

Diverse common analytical methods were used to perform MR analyses, including MR Egger, simple mode, inverse variance weighting (IVW), weighted mode, and weighted median. Of these, the IVW was the primary analytical method according to previously published literature (17, 18). MR Egger regression analysis was performed to detect the presence or absence of pleiotropy. If there was pleiotropy, the MR Egger method was applied (19). The MR Egger method is effective in addressing pleiotropy issues within genetic correlations and allowing for more accurate estimation of causal effects. It should be noted that “action = 2” should be selected in the “harmonise_data” function to reduce analytical bias caused by inconsistent alleles. The Cochrane Q value was used to detect the presence or absence of heterogeneity. If there was heterogeneity, the weighted median method was applied (20), because it took into account the weights of all individual SNP effects, thereby reducing the bias in estimation results. Additionally, the MR pleiotropy residual sum and outlier (MR-PRESSO) method was applied to detect the presence or absence of outliers. The MR analysis was done with the R package “TwoSampleMR” (version 0.5.7) (21).

The final conclusion was derived from the meta-analysis of MR results from the UKB and FinnGen databases, and was presented as odds ratios (OR) with corresponding 95% confidence intervals (95% CI). If there was significant heterogeneity between two cohorts (P < 0.05 or I^2^ > 50%), a random-effect model was employed, whereas a common-effect model was utilized if heterogeneity was not significant (P > 0.05 and I^2^ < 50%). The meta-analysis was done with the R package “meta” (version 6.5-0) (22). To be clear, four cancers were missing in the FinnGen database, including hepatocellular cancer, extrahepatic bile duct cancer, non-Hodgkin lymphoma, and hepatic bile duct cancer. Thus, MR analysis cannot be conducted on the causal relationships between THCA and these cancers, and the final conclusion was simply based on the UKB results.

### Investigation of shared genes and mechanisms between THCA and BLCA

RNA sequence data for THCA and BLCA were downloaded from the TCGA database. The TCGA THCA cohort contains 510 tumour samples and 58 normal samples, while the TCGA BLCA cohort contains 408 tumour samples and 19 normal samples. Searching for DEGs is the most commonly used research method for investigating potential pathogenic pathways in cancer. A 1.5-fold difference with a P-value < 0.05 was used as the criterion for screening DEGs. The DEG analysis was done with the R package “limma” (version 3.40.2), which calculated the statistical significance and fold change for each gene (23). Meanwhile, the WGCNA method was also applied, which clustered co-expressed genes into modules and facilitated the study of gene function and biological processes. The primary advantage of WGCNA lies in its capacity to convert gene data into biologically significant information, thereby providing clues for understanding the molecular mechanisms of diseases and discovering new biomarkers. The WGCNA analysis was done with the R package “wgcna” (version 1.72.5) (24). The overlapped genes between DEGs and WGCNA were considered key genes shared by THCA and BLCA.

Gene Ontology (GO) and Kyoto Encyclopedia of Genes and Genomes (KEGG) enrichment analysis were done with the R package “ClusterProfiler” (version 3.18.0) (25). This tool was primarily employed to assess whether a set of genes was significantly enriched within specific biological categories. Immune cell infiltration was estimated by the Microenvironment Cell Populations-counter (MCPcounter) algorithm from TIMER2.0 (http://timer.cistrome.org/). The is a method for estimating the relative abundance of various cellular subpopulations in tumor tissues based on transcriptomic data (26). It utilizes marker genes of specific cell types and linear regression models to estimate the content of different cell types in samples. Gene correlation analysis was performed using Gene Expression Profiling Interactive Analysis (GEPIA, http://gepia.cancer-pku.cn/) based on the Pearson method.

### Statistical analyses

Statistical analyses were done with R statistical software (version 4.3.1, The R Foundation for Statistical Computing, Vienna, Austria). A p-value less than 0.05 was considered statistically significant. The false discovery rate (FDR) method was applied to correct P-values.

## Results

After rigorous screening, a total of 19 SNPs were identified as instrumental variables referring to THCA (**Supplementary Table 4**). Subsequently, we investigated the causal relationships between THCA and cancers using the UKB and FinnGen cohorts.

### UKB cohort

**Figure 2:**
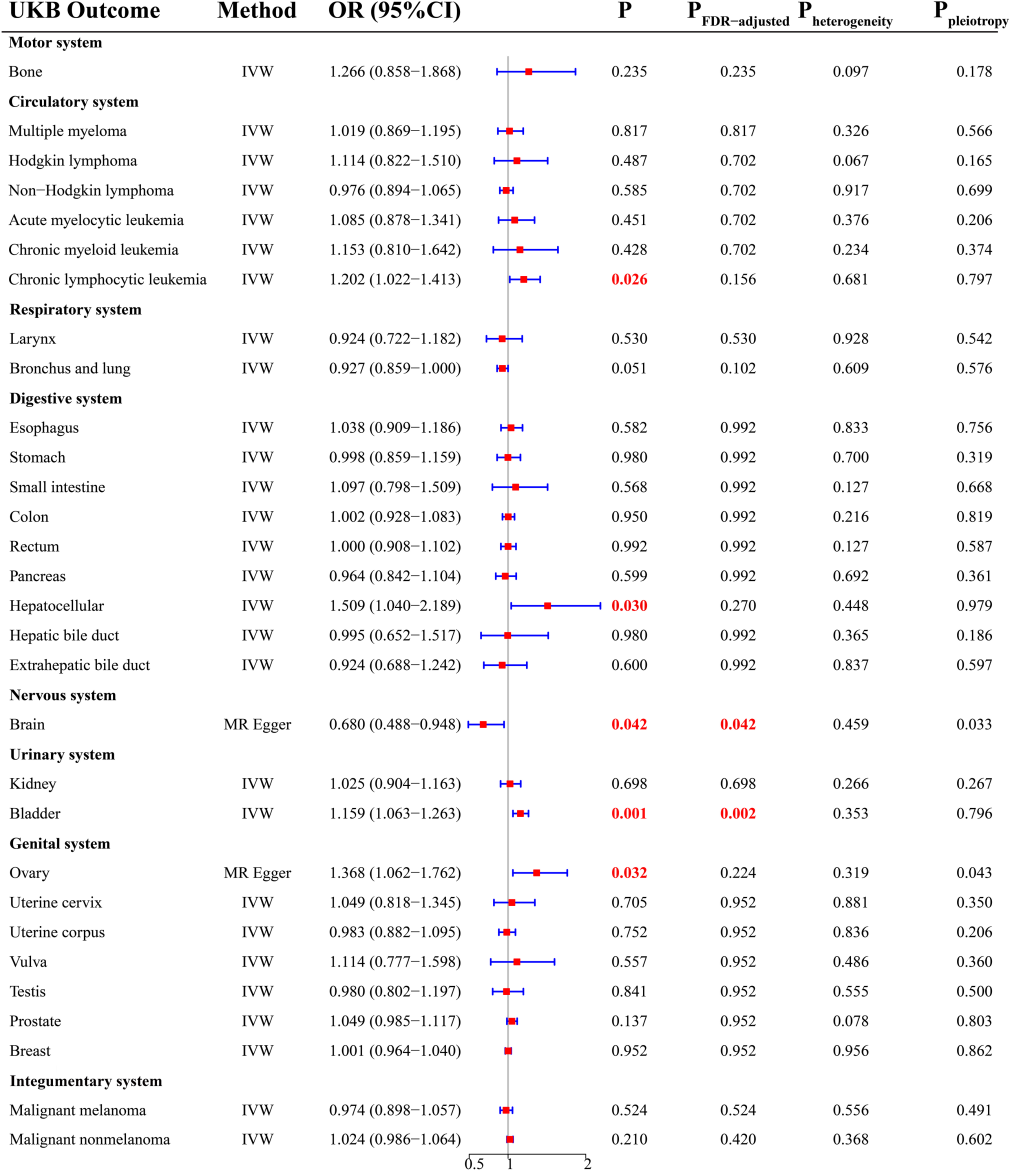
MR results from the UKB database.

There was a total of 30 cancer types in the UKB cohort. According to the MR analysis, there was a significant increase in the risk of four cancers (**Figure 2**), including chronic lymphocytic leukemia (OR = 1.202; 95% CI, 1.022-1.413; P = 0.026), hepatocellular cancer (OR = 1.509; 95% CI, 1.040-2.189; P = 0.030), bladder cancer (OR = 1.159; 95% CI, 1.063-1.263; P = 0.001), and ovarian cancer (OR = 1.368; 95% CI, 1.062-1.762; P =0.032). After FDR correction, THCA was still positively related to BLCA (P = 0.002). For cancers with P < 0.05 and P_FDR-adjusted_ > 0.05, we considered a potential causal relationship between THCA and them. In addition, THCA had a negative causality with brain malignancy (**Figure 2**, OR = 0.680; 95% CI, 0.488-0.948; P =0.042), indicating the protection of THCA against brain malignancy.

Sensitivity analysis showed that there was potential pleiotropy for brain malignancy (**Figure 2**, P = 0.033) and ovarian cancer (**Figure 2**, P = 0.043). The MR outcomes yielded by the MR Egger method were selected. There was no evidence of the presence of heterogeneity and outliers. Detailed MR results were presented in **Supplementary Table 5**.

### FinnGen cohort

**Figure 3:**
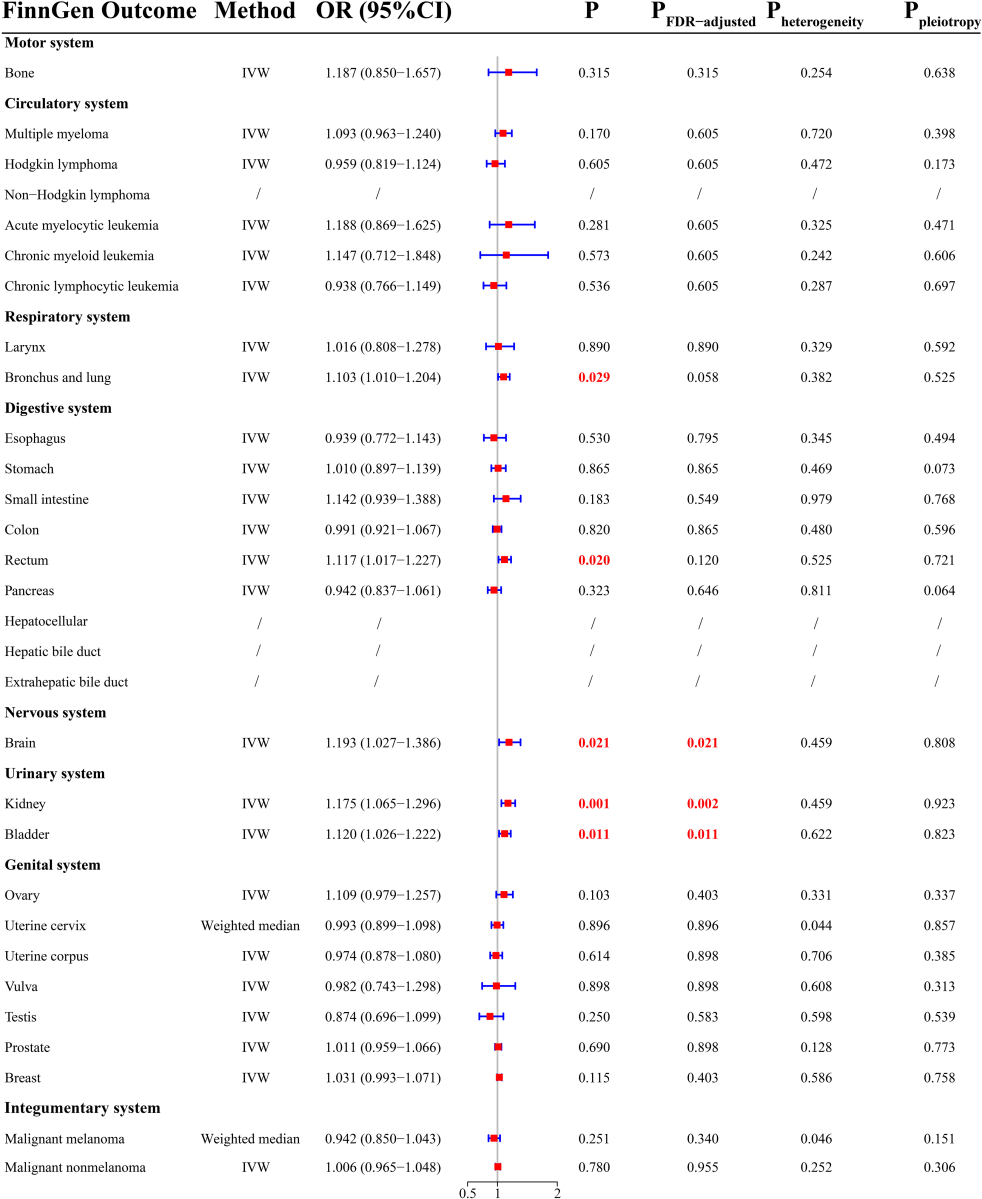
MR results from the FinnGen database.

There was a total of 26 cancer types in the FinnGen cohort. MR analysis demonstrated that THCA had a positively causal effect on five cancers (**Figure 3**), including malignancy of bronchus and lung (OR =1.103; 95% CI, 1.010-1.204; P = 0.029), rectal cancer (OR = 1.117; 95% CI, 1.017-1.227; P = 0.020), brain malignancy (OR = 1.193; 95% CI, 1.027-1.386; P = 0.021), kidney cancer (OR = 1.175; 95% CI, 1.065-1.296; P = 0.001), and bladder cancer (OR = 1.120; 95% CI, 1.026-1.222; P = 0.011). After FDR correction, THCA was significantly related to an increased risk of brain malignancy (P = 0.021), kidney cancer (P = 0.002), and bladder cancer (P = 0.011). There was a potential causal relationship between THCA and malignancy of bronchus and lung (P_FDR-adjusted_ = 0.058) and rectal cancer (P_FDR-adjusted_ = 0.120). Surprisingly, there were contradictory outcomes regarding the causal relationship between THCA and brain malignancy in the UKB and FinnGen cohorts.

Sensitivity analysis showed that there was potential heterogeneity for cervical cancer (**Figure 3**, P = 0.044) and malignant melanoma (**Figure 3**, P = 0.046). The MR outcomes yielded by the weighted median method were selected. There was no evidence of pleiotropy. MR-PRESSO indicated the presence of outliers (rs34393407 and rs7027030) for malignancy of bronchus and lung, and the results presented above were based on data with outliers removed. Detailed MR results were presented in **Supplementary Table 6**.

### Meta-analysis

**Figure 4:**
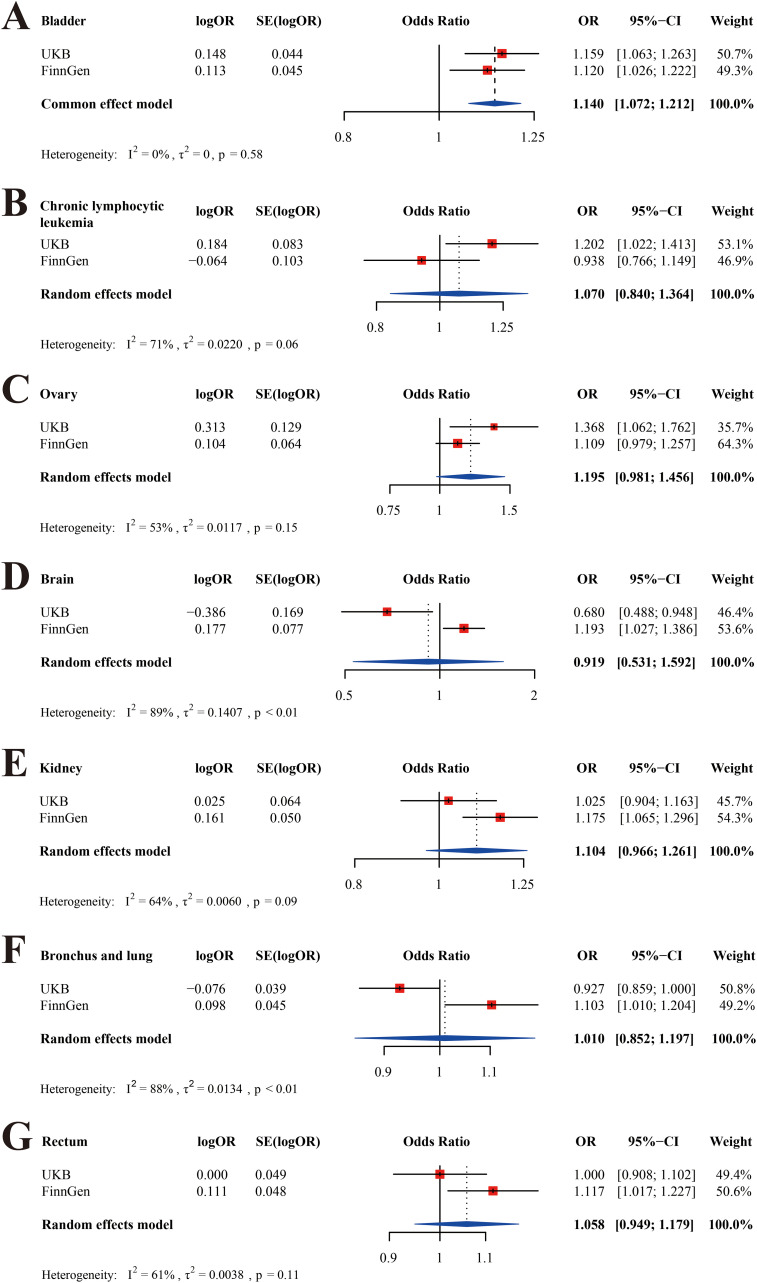
Meta-analyses of the UKB and FinnGen cohorts. **(A)** bladder cancer; **(B)** chronic lymphocytic leukemia; **(C)**ovarian cancer; **(D)** brain malignancy; **(E)** kidney cancer; **(F)** malignancy of bronchus and lung; **(G)** rectal cancer.

Meta-analysis was conducted for all types of cancer with P < 0.05 in the UKB or FinnGen database, including chronic lymphocytic leukemia, bladder cancer, ovarian cancer, brain malignancy, kidney cancer, rectal cancer, and malignancy of bronchus and lung (**Figure 4**). To be clear, hepatocellular cancer was not included owing to the lack of data in the FinnGen database. We found that THCA was positively related to BLCA after the meta-analysis (**Figure 4A**, OR = 1.140; 95% CI, 1.072-1.212; P < 0.001). There was no significant causality between THCA and the other cancers (**Figures 4B–G**, P > 0.05).

### Shared genes and potential mechanisms between THCA and BLCA

**Figure 5:**
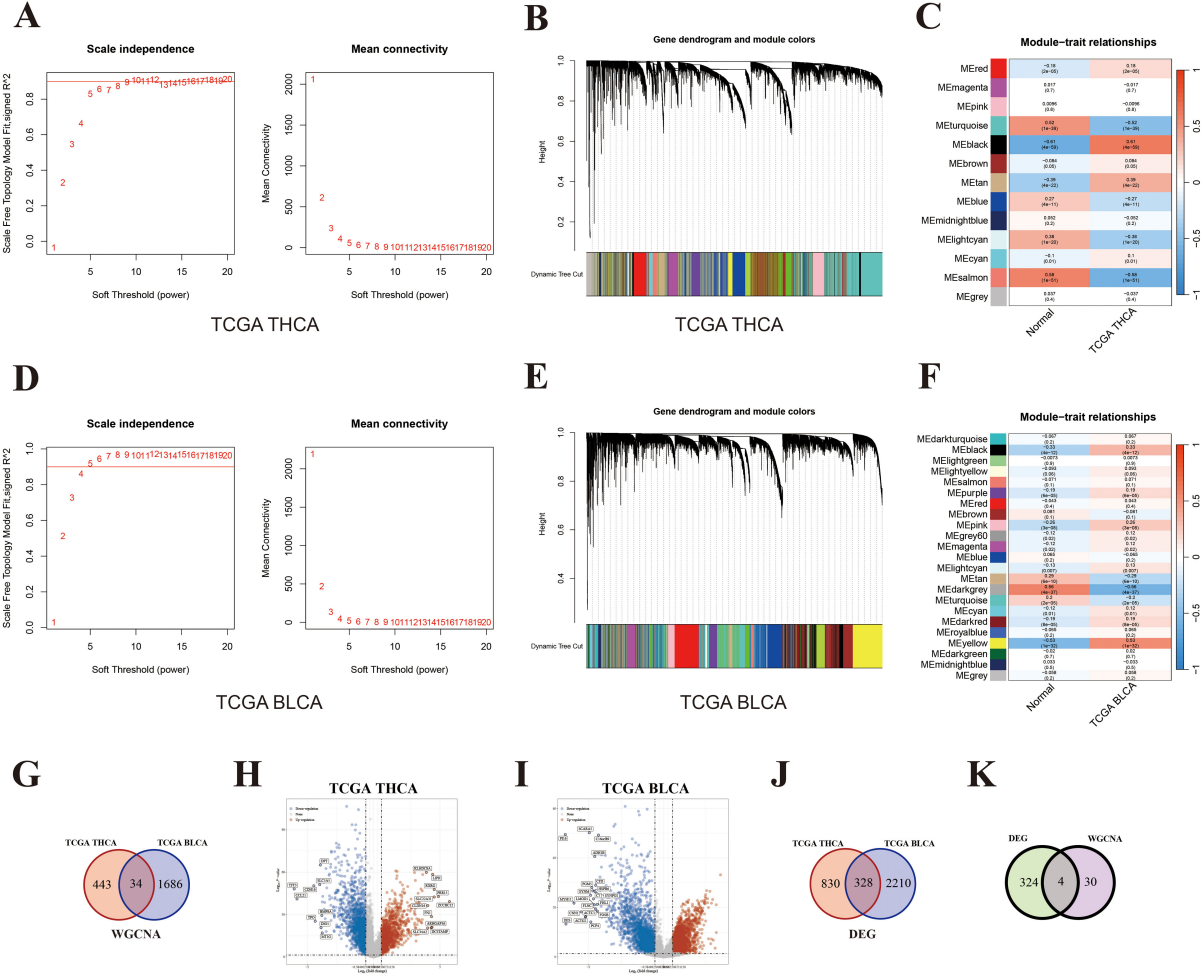
Key genes shared by both THCA and BLCA. **(A)** Softpower of WGCNA for THCA; **(B)** Module correlation plot of WGCNA for THCA; **(C)** Module–trait relationships for THCA; **(D)** Softpower of WGCNA for BLCA; **(E)** Module correlation plot of WGCNA for BLCA; **(F)** Module–trait relationships for BLCA; **(G)** Venn diagram of hub genes shared by THCA and BLCA; **(H)** Volcano map of DEGs between THCA and normal thyroid tissues; **(I)** Volcano map of DEGs between BLCA and normal bladder tissues; **(J)** Venn diagram of DEGs shared by THCA and BLCA; **(K)** Venn diagram of key genes shared by THCA and BLCA based on the results from WGCNA and DEGs.

First, the WGCNA analysis was performed to identify co-expressed genes between THCA and BLCA. For THCA, the condition for softpower was set as 6 (**Figure 5A**) and a total of 13 modules were generated (**Figures 5B, C**). The ME black module (including 477 genes) exhibited the strongest positive correlation with THCA (**Figure 5C**; **Supplementary Table 7**, r = 0.61, P = 4 × 10^–59^). Similarly, the condition for softpower was set as 4 (**Figure 5D**) and a total of 23 modules were generated for BLCA (**Figures 5E, F**). The ME yellow module (including 1720 genes) exhibited the strongest positive correlation with BLCA (**Figure 5F**; **Supplementary Table 8**, r = 0.53, P = 1 × 10^–32^). The overlapped genes in the black module of THCA and yellow module of BLCA were considered as hub genes shared by THCA and BLCA, whose number was 34 (**Figure 5G**; **Supplementary Table 9**).

Next, DEGs were further screened (difference > 1.5-fold change; P < 0.05). A total of 1158 and 2538 up-regulated genes were identified in the TCHA and BLCA cohort, respectively (**Figures 5H, I**; **Supplementary Tables 10**, **11**). The number of overlapped genes between TCHA and BLCA was 328 (**Figure 5J**; **Supplementary Table 12**). Ultimately, we took the intersection of WGCNA and DEG results, and identified four shared genes between TCHA and BLCA, including WNT3, FAM171A2, MLLT11, and ULBP1 (**Figure 5K**). Additionally, we adopted an alternative analytical method by performing DEG analysis directly within the modules significantly associated with the diseases (THCA or BLCA), and also identified the same four shared genes (**Supplementary Figure 1**).

**Figure 6:**
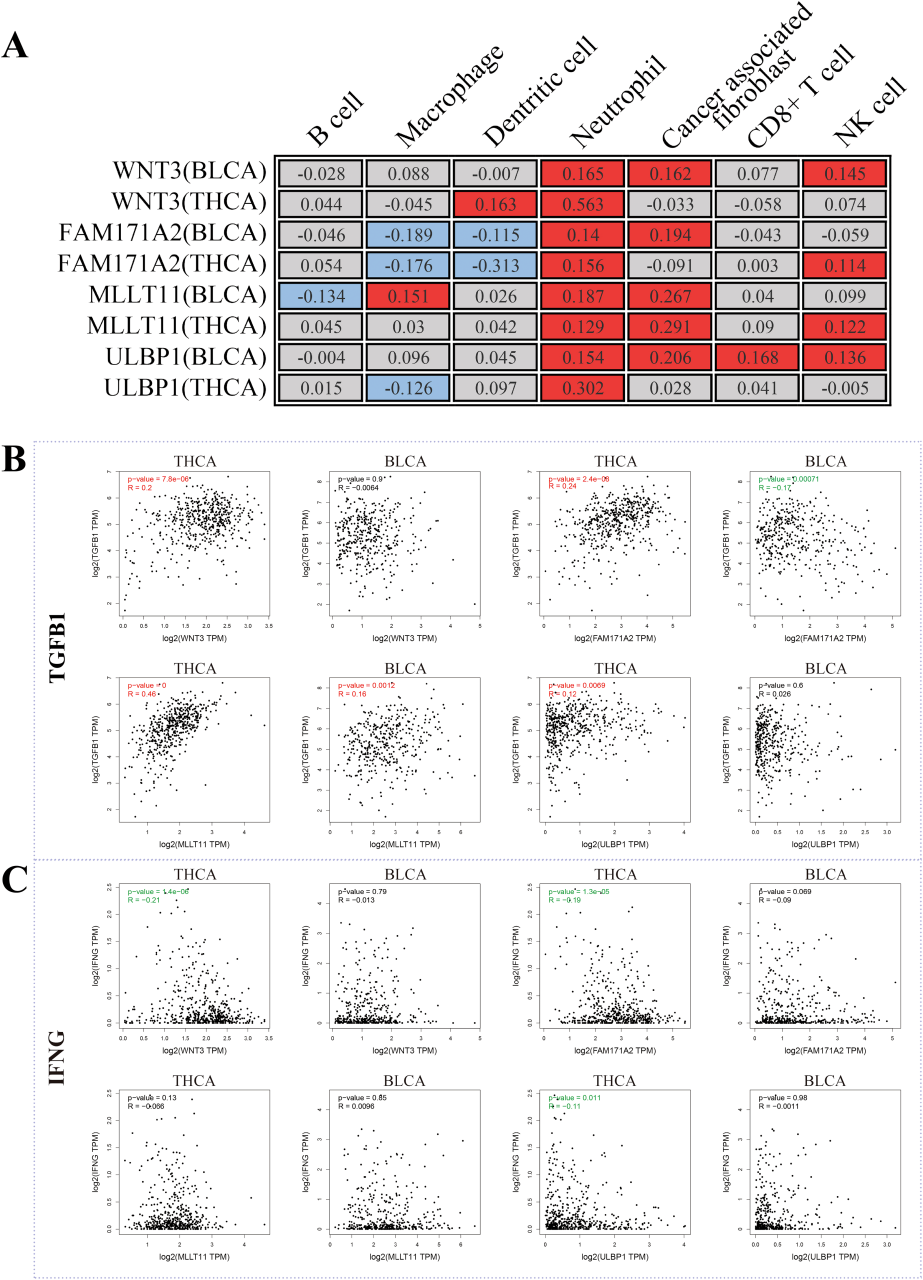
Potential mechanisms shared by both THCA and BLCA. **(A)** The correlation between shared genes and immunoinfiltration; **(B)** The correlation between shared genes and TGFB1 (a key marker of N2 neutrophil infiltration); **(C)** The correlation between shared genes and IFNG (a key marker of N1 neutrophil infiltration).

Using these four shared genes, we conducted GO and KEGG enrichment analysis. However, there were no significant GO terms or KEGG pathways identified. Thus, we further investigated whether these genes had an effect on immune cell infiltration. According to the “MCPcounter” algorithm, these four genes were indeed significantly associated with immune cell infiltration (**Figure 6A**). Among different immune cells, all four genes in both THCA and BLCA showed significantly positive correlations with neutrophils (P < 0.05), indicating that these genes may exert biological functions by increasing the infiltration of neutrophils. Increasing literature has indicated that neutrophils could be classified into two functional categories: anti-tumor N1 neutrophils and pro-tumor N2 neutrophils (27). The former was dominated by the type I IFN signaling pathway, while the latter was dominated by the TGFβ signaling pathway. Therefore, we evaluated the relationships between shared genes and key markers of these pathways. TGFB1, a key gene in the TGFβ signaling pathway, was shown to be positively correlated with the majority of shared genes (**Figure 6B**). Conversely, IFNG, a key gene in the type I IFN signaling pathway, showed a negative or no correlation with shared genes (**Figure 6C**). These findings indicated that shared genes between THCA and BLCA were positively associated with increased infiltration of N2 neutrophils. Overall, it is reasonable to speculate that THCA may increase the risk of secondary BLCA through augmentation of N2 neutrophil infiltration.

## Discussion

The MR analysis provided us with a reliable tool for assessing the risk of SPMs in THCA patients. Using the GWAS summary datasets with a large sample size, we found that THCA caused an increase in risk for a considerable proportion of cancer types, including chronic lymphocytic leukemia, BLCA, ovarian cancer, kidney cancer, rectal cancer, and malignancy of bronchus and lung, and hepatocellular cancer. These findings were generally consistent with a number of epidemiological studies (28, 29). However, after adjusting P-values or conducting the meta-analysis, the elevation in risk for most SPMs was no longer statistically significant with the exception of BLCA. This suggests that most SPMs following THCA are likely caused by factors other than genetic variants. However, more researches are necessary to study the risk of specific-site cancers following THCA, with the aim of formulating evidence-based monitoring guidelines and reducing the overall mortality from THCA.

Our study demonstrated that THCA was significantly associated with an increased risk of BLCA, which was confirmed by several large-sample observational studies. A retrospective analysis from the Taiwan Cancer Registry indicated a notable increase in the risk of multiple malignancies including BLCA (30). Upon stratification by age of diagnosis and follow-up duration, it was observed that patients with younger age (less than 50 years) exhibited a higher susceptibility to SPMs including BLCA, particularly within the initial five-year period after diagnosis. Another South Korea study also demonstrated that there was a greater risk of BLCA (standardized incidence ratio [SIR]: 1.54) in patients with a history of THCA (31). Furthermore, it was observed that frequent (2 times or more) medical radiation exposure from computed tomography (CT) or positron emission tomography-CT (PET-CT) was an independent risk factor for developing a secondary BLCA in female patients with THCA, but not in males. Additionally, RAI therapy did not promote the risk of secondary BLCA in THCA patients of both genders (P = 0.397). These indicated that only a small portion of the elevated risk of BLCA could be attributed to radiation exposure. Akslen et al. (32) conducted a nationwide study with a follow-up period of up to 30 years in Norway, and found a significantly increased risk of secondary urogenital cancers (e.g. BLCA and testis cancer) in male patients with THCA. However, they cannot explain this phenomenon. Our study provided evidence for the causal relationship between THCA and BLCA from the genetic perspective, and facilitated a deeper understanding of potential associations between them. Using an integrated bioinformatics approach, we identified four shared genes between THCA and BLCA. MLLT11 (AF1q), as an oncogenic factor in the thyroid tumorigenesis, also played a significant role in the onset and progression of BLCA (33–35). Further analysis indicated that an increased infiltration of N2 neutrophils may be a key factor in the elevated risk of BLCA secondary to THCA. Numerous studies indicated that N2 neutrophil infiltration played a crucial role in the progression of malignant tumors including BLCA and THCA (36–40). In particular, there was a distinct subgroup of neutrophils called tumor-associated neutrophils (TANs), which facilitated the formation and maintenance of an immunosuppressive microenvironment by producing immunosuppressive cytokines (e.g. IL-10 and TGF-β) and inhibiting effector T cells (41, 42). Moreover, neutrophil-to-lymphocyte ratio, a common systemic inflammatory marker, demonstrated high prognostic values in both THCA and BLCA patients (43–46), which might be a simple and feasible method of specific surveillance for the BLCA risk during the postoperative follow-up course of THCA patients. Currently, the latest American Thyroid Association (ATA) guidelines (2015) only offered feasible follow-up procedures for recurrence and metastasis of THCA, but did not provide clear follow-up recommendations for SPMs (47). Our findings laid the foundation for personalized treatment of patients with THCA. We recommended implementing targeted screening for secondary BLCA during the long-term follow-up of THCA, such as tumor cell detection in urine annually and cystoscopy every two to three years.

However, the findings from some large-sample studies were not consistent with ours. A population-based Surveillance, Epidemiology, and End Results (SEER) analysis did not detect an increased risk of BLCA following a diagnosis of THCA (7, 10). Another international study including 39,002 individuals also yielded negative results (48). There are two main reasons for the inconsistencies. On one hand, numerous potential factors may lead to the inconsistency, including the kind and duration of treatment, follow-up approaches, and environmental variance. On the other hand, various populations exhibit significant differences in the genetic and molecular background.

There were a few limitations in the present study. First, all GWAS summary datasets were derived from European populations, which may limit the potential generalization of our conclusions. Second, bioinformatics approaches were utilized to identify potential mechanisms shared by THCA and BLCA, yet experimental validation was not performed. Third, we did not differentiate between different pathological subtypes of THCA, limiting its implications for personalized patient management. Fourth, RAI and radiation therapy were possible confounders for SPMs in patients with THCA. However, there are currently no SNPs that have been definitively linked in a causal relationship to RAI or radiation therapy, potentially leading to some bias.

In summary, we systematically investigated the causal relationships between TCHA and SPMs, and found that THCA may increase the risk of secondary BLCA through augmentation of N2 neutrophil infiltration. This provided the basis for optimizing the follow-up management of THCA patients.

## Data Availability

The original contributions presented in the study are included in the article/**Supplementary Material**. Further inquiries can be directed to the corresponding authors.

## Supplementary material

Supplementary Figure 1 Shared genes identified by performing DEG analysis directly within the modules significantly associated with the disease.

Supplementary Table 1 Removed IVs.

Supplementary Table 2 Outcomes from the UKB database.

Supplementary Table 3 Outcomes from the FinnGen database.

Supplementary Table 4 IVs for THCA.

Supplementary Table 5 Causal effects of THCA on pan-cancer and sensitivity analyses in the UKB database.

Supplementary Table 6 Causal effects of THCA on pan-cancer and sensitivity analyses in the FinnGen database.

Supplementary Table 7 Detailed gene list in modules with the highest positive correlation coefficients for THCA.

Supplementary Table 8 Detailed gene list in modules with the highest positive correlation coefficients for BLCA.

Supplementary Table 9 Hub genes shared by THCA and BLCA.

Supplementary Table 10 Detailed list of DEGs in THCA.

Supplementary Table 11 Detailed list of DEGs in BLCA.

Supplementary Table 12 DEGs shared by THCA and BLCA.
