# Supplementary figures and images for: Causal effect of thyroid cancer on secondary primary malignancies: findings from the UK Biobank and FinnGen cohorts
Source: Front Immunol. 2024 Sep 26;15:1434737. doi: 10.3389/fimmu.2024.1434737 (PMC11464368; doi:10.3389/fimmu.2024.1434737)

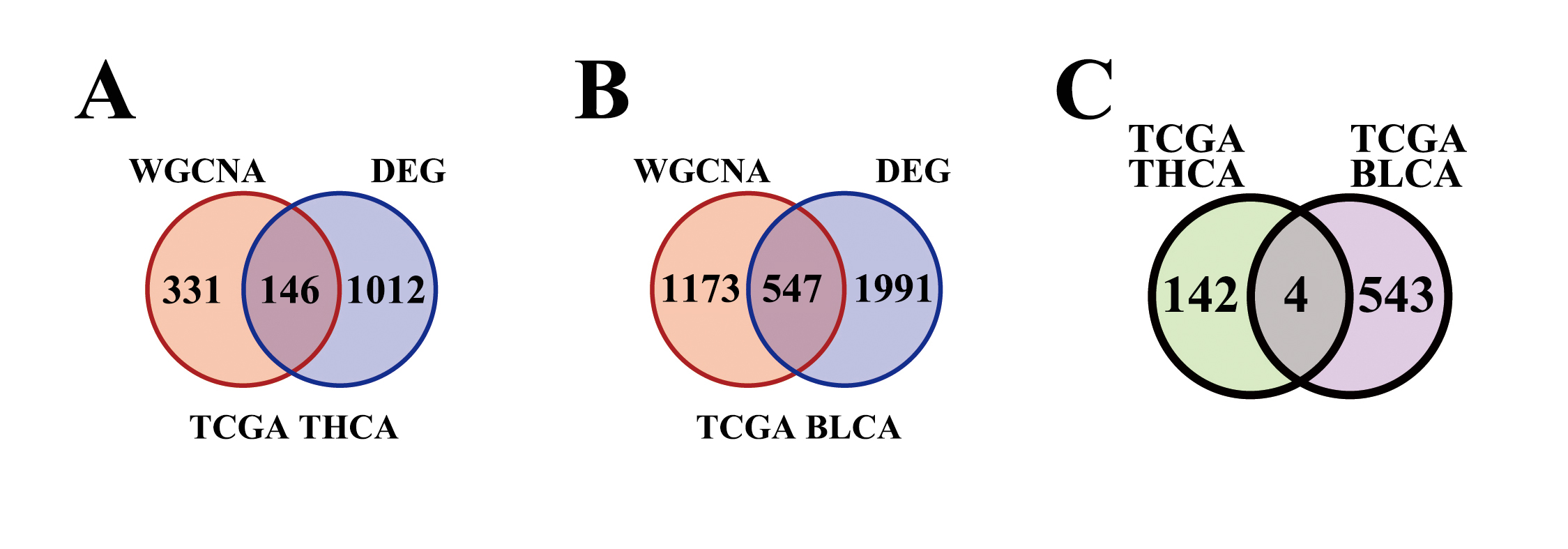

Supplement: Supplementary Figure 1 — Shared genes identified by performing DEG analysis directly within the modules significantly associated with the disease. [file Image1.jpeg]
